# Receptor Complementation and Mutagenesis Reveal SR-BI as an Essential HCV Entry Factor and Functionally Imply Its Intra- and Extra-Cellular Domains

**DOI:** 10.1371/journal.ppat.1000310

**Published:** 2009-02-20

**Authors:** Marlène Dreux, Viet Loan Dao Thi, Judith Fresquet, Maryse Guérin, Zélie Julia, Géraldine Verney, David Durantel, Fabien Zoulim, Dimitri Lavillette, François-Loïc Cosset, Birke Bartosch

**Affiliations:** 1 Université de Lyon, UCB-Lyon1, IFR128; INSERM, U758; Ecole Normale Supérieure de Lyon, Lyon, France; 2 INSERM, U551, Paris, France; 3 Université de Lyon, UCB-Lyon1, IFR62; INSERM, U871; Hospices civils de Lyon (HCL), Lyon, France; Mount Sinai School of Medicine, United States of America

## Abstract

HCV entry into cells is a multi-step and slow process. It is believed that the initial capture of HCV particles by glycosaminoglycans and/or lipoprotein receptors is followed by coordinated interactions with the scavenger receptor class B type I (SR-BI), a major receptor of high-density lipoprotein (HDL), the CD81 tetraspanin, and the tight junction protein Claudin-1, ultimately leading to uptake and cellular penetration of HCV *via* low-pH endosomes. Several reports have indicated that HDL promotes HCV entry through interaction with SR-BI. This pathway remains largely elusive, although it was shown that HDL neither associates with HCV particles nor modulates HCV binding to SR-BI. In contrast to CD81 and Claudin-1, the importance of SR-BI has only been addressed indirectly because of lack of cells in which functional complementation assays with mutant receptors could be performed. Here we identified for the first time two cell types that supported HCVpp and HCVcc entry upon ectopic SR-BI expression. Remarkably, the undetectable expression of SR-BI in rat hepatoma cells allowed unambiguous investigation of human SR-BI functions during HCV entry. By expressing different SR-BI mutants in either cell line, our results revealed features of SR-BI intracellular domains that influence HCV infectivity without affecting receptor binding and stimulation of HCV entry induced by HDL/SR-BI interaction. Conversely, we identified positions of SR-BI ectodomain that, by altering HCV binding, inhibit entry. Finally, we characterized alternative ectodomain determinants that, by reducing SR-BI cholesterol uptake and efflux functions, abolish HDL-mediated infection-enhancement. Altogether, we demonstrate that SR-BI is an essential HCV entry factor. Moreover, our results highlight specific SR-BI determinants required during HCV entry and physiological lipid transfer functions hijacked by HCV to favor infection.

## Author Summary

More than 180 million people are chronically infected by hepatitis C virus (HCV), a leading cause of liver failure and cancer, stimulating the need to fully define the biology of HCV infection for developing novel and effective therapeutics. During the first steps of infection, the virus is taken up and penetrates hepatocytes. HCV entry is thought to be a coordinated multi-step process mediated by specific factors, including CD81, Claudin-1, and the scavenger receptor BI (SR-BI). Whereas the involvement of CD81 and Claudin-1 was demonstrated by rendering susceptible cells that are otherwise refractory, SR-BI complementation assays were lacking, raising questions as to its functions during HCV entry. Here, we identify one hepatoma rat cell line, in which SR-BI complementation assay and targeted mutagenesis could be performed. We therefore demonstrate that SR-BI is an essential HCV entry factor. Our results shed light on SR-BI intracellular domain functions in HCV entry, and, further, emphasize the remarkable capacity of HCV to hijack the lipid transfer function of SR-BI, hence favoring infection.

## Introduction

Hepatitis C virus (HCV) infection is a leading cause of chronic liver disease world-wide. With 180 million persistently infected people, chronic hepatitis C infection, which induces end-stage liver disease such as liver cirrhosis and hepatocellular carcinoma (HCC), represents a major public health problem of high socio-economic impact [1]. However, treatment options for chronic hepatitis C are limited, and a vaccine for prevention against HCV infection is not available. HCV is a positive strand RNA enveloped virus from the *Flaviviridae* family. Viral attachment and entry—representing the first encounter of the virus with the host cell—are major targets of adaptive host cell defenses. Detailed understanding of the HCV entry process should offer interesting opportunities for development of novel therapeutic strategies to prevent or cure HCV infection.

HCV entry is thought to be a multi-step process (reviewed in [2],[3],[4]). The interactions between envelope glycoproteins and glycosaminoglycans might contribute to the primary binding of virus particles to host cells. Because of the association of HCV with low-density lipoproteins (LDL) in serum of infected patients [5], the LDL receptor (LDLr) has also been proposed as an alternative capture receptor [6]. Following this initial engagement, the scavenger receptor class B type I (SR-BI) [7], the CD81 tetraspanin [8] and the tight junction protein Claudin-1 (CLDN1) [9] may contribute to uptake and cellular penetration of HCV in a clathrin-dependent manner [10],[11]. Using HCVpp and HCVcc infection assays as well as *in vitro* membrane fusion assays, HCV entry was shown to occur in a pH-dependent manner [12],[13],[14],[15],[16], through endocytosis of the viral particles [10],[11]. Like other *Flaviviridae* [17], the low endosomal pH may induce conformational rearrangement of HCV glycoproteins, leading to fusion of the viral membrane with that of the endosome.

The exact function of these molecules, and particularly SR-BI, in HCV infection is still enigmatic. SR-BI, also called CLA-1, was originally defined as a class B scavenger receptor [18] in a family that includes CD36, LIMPII and SR-BII, an SR-BI isoform with an alternate cytoplasmic tail [19]. SR-BI mediates binding and lipid transfer from different classes of lipoproteins [20], particularly high-density lipoprotein (HDL), accounting for its multiple functions in cholesterol metabolism such as removal of peripheral unesterified cholesterol, steroidogenesis and bile acid synthesis and secretion. SR-BI stimulates the bi-directional flux of free cholesterol (FC) between cells and lipoproteins, an activity that may be responsible for net cholesterol efflux from peripheral cells as well as the rapid hepatic clearance of FC from plasma HDL. In hepatic cells, SR-BI also mediates the selective uptake of cholesteryl ester (CE) from HDL, a process by which HDL CE is taken into the plasma membrane without degradation of the HDL particle [21]. Through lipid uptake, SR-BI increases cellular cholesterol mass and alters cholesterol distribution in plasma membrane domains [22],[23].

SR-BI mediates binding of the E2 [7], one of the two glycoproteins exposed at the surface of HCV particles, and, as a multiligand lipoprotein receptor, can also induce binding of HCV associated to LDL [24]. Intriguingly, we, and others, have demonstrated that HDL enhances infectivity of HCVpp and HCVcc [25],[26],[27],[28],[29],[30]. This original mechanism is controlled by the HCV glycoproteins, and, more particularly, by conserved residues of the hypervariable region-1 (HVR1) [25],[29], a 27 amino-acid peptide located at the amino-terminus of E2. HDL-mediated enhancement of infection clearly involves SR-BI but this occurs neither through a direct binding of HDL to HCV particles nor through increase of HCV binding to SR-BI [25],[27],[29]. Since SR-BI locally increases cholesterol content of cell membranes by mediating lipid transfer from HDL, it has been proposed that HCV may exploit SR-BI physiological function to achieve its entry processes [25],[29]. However, direct evidence is missing that, by providing a docking port to HCV particle and/or by modulating post-binding events, SR-BI favors infection. Indeed, in contrast to CD81 and CLDN1 whose implications during HCV entry have been unambiguously demonstrated by mutational analysis in cells that were rendered susceptible to HCV entry upon their ectopic expression [9],[12],[13], the importance of SR-BI has only been addressed indirectly because of lack of cells in which similar complementation assays could be performed. Here we identified two cell types, of rat and human origins, that supported efficient HCV entry upon ectopic expression of SR-BI. Through the design of SR-BI mutants, i.e., in the extracellular and the cytoplasmic domains, we unravel important features of SR-BI functions regarding its involvement during HCV entry.

## Results

### Susceptibility to HCVpp and HCVcc entry upon ectopic expression of HCV receptors

**Figure 1:**
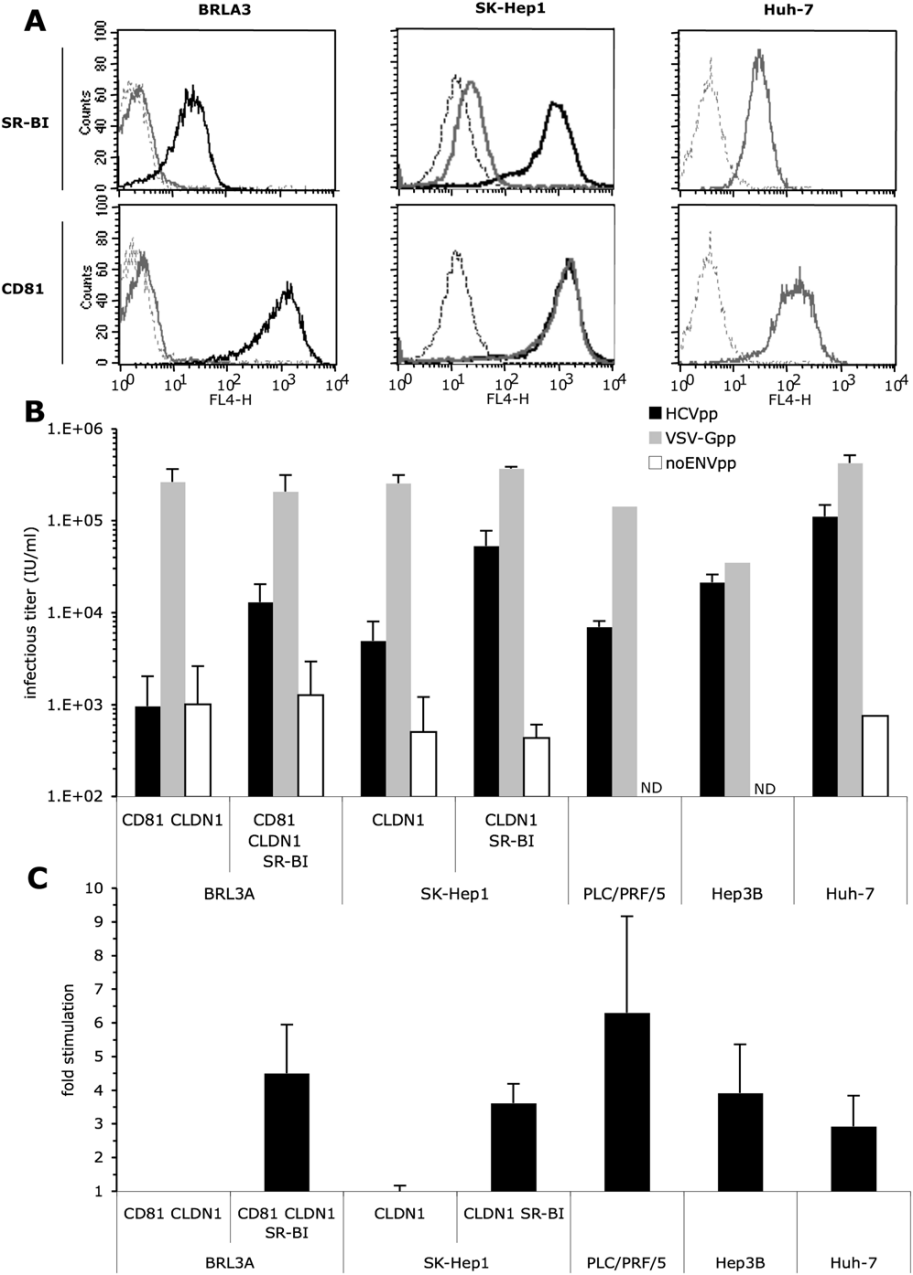
HCVpp entry upon ectopic hSR-BI co-expression with other HCV receptors. (A) Endogenous and/or ectopic expression of hSR-BI and hCD81 in BRL3A, SK-Hep1, and Huh-7 cells was determined by flow cytometry. Parental cells were stained with hSR-BI (CLA-1 mAb, upper panel) or hCD81 (JS81 mAb, lower panel) antibodies (gray lines). The background of fluorescence was provided by staining the cells with the secondary antibodies only (dotted lines). Ectopic expression of hSR-BI in BRL3A and SK-Hep1 cells or of hCD81 in BRL3A cells was determined using the same antibodies (black lines). The results are representative of three independent experiments. (B) Results of HCV entry assays on BRL3A and SK-Hep1 cells ectopically expressing the indicated HCV receptors, and on PLC/PRF/5, Hep3B, and Huh-7 human hepatoma cells, endogenously expressing CD81, CLDN1, and SR-BI, using HCV pseudo-particles harboring H77-E1E2 glycoproteins (HCVpp), control viral particles harboring the VSV-G glycoprotein (VSV-Gpp; diluted 1/100), or no glycoprotein (noENVpp). The viral particles were produced in cell culture media devoid of serum lipoproteins. Results display average infectious titers, expressed as GFP IU/ml (mean±SD; n = 3). ND, not determined. (C) HCV entry assays using HCVpp produced in serum-free medium in the presence of 6 µg/ml cholesterol-HDL. The results show the fold increases of infection (mean±SD; n = 3) determined by calculating the ratios between average infectious titers determined in the presence or absence of HDL. No changes of infectivity with VSV-Gpp control particles were detected under these experimental conditions (data no shown), as reported previously [25].

Challenging the notion of liver tropism of HCV, most HCV receptors isolated so far are broadly expressed in different tissues, including those in which HCV does not replicate [2],[3],[4]. Particularly, the broadness of SR-BI expression makes difficult the investigation of the functional properties of this molecule in HCV infection, owing to the paucity of SR-BI-negative human cell lines in which susceptibility to HCV entry could be obtained upon ectopic expression. Furthermore, efficient SR-BI down-regulation in susceptible cell types, such as Huh-7 cells, is difficult to achieve without compromising cell viability (MD, DL, BB and FLC, data not published) and has raised different results between studies [14],[25],[29],[31],[32]. To overcome these difficulties, we screened a panel of human and rodent cell lines for absence or low SR-BI expression (Table 1). Aiming to design functional complementation assays, we ectopically expressed in these cells HCV receptors by transduction with a set of selectable retroviral vectors encoding human CD81, CLDN1 or SR-BI and, upon appropriate selection, we challenged these cells with HCV pseudo-particles (HCVpp). We detected one human liver endothelial cell line, SK-Hep1, and one rat hepatocarcinoma cell line, BRL3A, that became susceptible to entry with HCVpp-H77 (Table 1) and with HCVpp of other genotypes (Figure S1C) upon ectopic expression of SR-BI, provided hCD81 and hCLDN1 were also co-expressed, endogenously or ectopically (Figure 1 and Figure S1). While non hepato-carcinoma cells of human origin like e.g., 293T cells, have been rendered susceptible to HCV entry upon ectopic expression of hCLDN1 [9], this is the first report of a non human cell line that can be functionally complemented by HCV receptors, allowing efficient HCV entry.

**Table 1:** HCVpp entry in receptor-complemented target cells.

Cell Lines	Tissue	HCVpp Entry^a^	hCD81^b^	hCLDN1^c^	hSR-BI^d^
Huh-7	Human hepatoma	++	+	+	+
PLC/PRF/5	Human hepatoma	+	++	+	+
Hep3B	Human hepatoma	+	+	+	+
CHO	Chinese hamster ovary	−	−	−	−
CHO-CD81-CLDN1-SR-BI	Chinese hamster ovary	−	++	++	++
MDCK	Dog kidney epithelium	−	−	−	−
MDCK-CD81-CLDN1-SR-BI	Dog kidney epithelium	−	++	++	++
Hepa1.6	Mouse hepatoma	−	−	−	−
Hepa1.6-CD81-CLDN1-SR-BI	Mouse hepatoma	−	+	+	++
XC	Rat fibrosarcoma	−	−	−	−
XC-CD81-CLDN1-SR-BI	Rat fibrosarcoma	−	+	+	+
BRL3A	Rat hepatoma	−	−	−	−
BRL3A-CD81-CLDN1-SR-BI	Rat hepatoma	+	+	+	+
SK-Hep1	Human liver endothelium	±	+	−	±
SK-Hep1-CD81-CLDN1-SR-BI	Human liver endothelium	+	+	++	++

a HCVpp entry of genotype 1a (H77) harbouring the GFP marker gene. (++), titres higher than 10^5^ IU/ml; (+), titres between 10^3^ and 10^5^ IU/ml; (±), titres between 10^2^ and 10^3^ IU/ml; (−), titres lower than 10^2^ IU/ml, which corresponds to the threshold of detection of infected cells by FACS analysis.

b Detection of human CD81 using JS-81 antibody on the surface of the indicated cells by flow cytometry. (−), MFI (mean fluorescent intensity) shift of 1; (+), MFI shift between 1 and 50; (++), MFI shift over 50.

c Detection of human Claudin-1 (CLDN1) in lysates of the indicated cells by immuno-blotting with mouse anti-Claudin-1 antibodies (Interchim). (−), signal intensity 5-fold lower than that detected in Huh-7 cells; (+), signal intensity between 5-fold lower and 5-fold higher than that detected in Huh-7 cells; (++), signal intensity 5-fold higher than that detected in Huh-7 cells.

d Detection of human SR-B1 using the CLA-1 antibody on the surface of the indicated cells by flow cytometry. (−), MFI shift of less than 2; (±), MFI shift between 2 and 6; (+), MFI shift between 6 and 20; (++), MFI shift over 20.

Over-expression of hSR-BI in SK-Hep1-CLDN1 cells that express very low endogenous SR-BI levels (Figure 1A and Figure S1A) resulted in ca. 10-fold increased HCVpp titers, i.e., titers that were only 2-fold lower than those obtained in Huh-7 cells (Figure 1B and Figure S1D). In contrast, no or undetectable expression of endogenous rat SR-BI could be detected by immunoblotting and by RT-qPCR in BRL3A cells (Figure S1A and S1B, Protocol S1). Furthermore, no or hardly detectable HCV entry could be found in BRL3A cells ectopically expressing hSR-BI only or expressing both hCD81 and hCLDN1 (BRL3A-CD81-CLDN1 cells), despite full entry susceptibility of control pseudo-particles pseudotyped with VSV-G glycoprotein (Figure 1B and Figure S1D). Expression of hSR-BI in BRL3A-CD81-CLDN1 cells allowed HCVpp entry at titers similar to those detected in PLC/PRF/5 and Hep3B human hepato-carcinoma cells (Figure 1B), used in previous reports [12],[13],[33],[34], and ca. 8-fold lower than those obtained with Huh-7 cells (Figure 1B), which are the most susceptible to HCV entry [12],[13],[14],[33]. Of note, co-expression of hCD81, hCLDN1 and/or hSR-BI did not modify total (Figure S1A) or cell surface (Figure S2) expression levels of either of these entry factors. Hence, our results unambiguously demonstrated for the first time that expression of SR-BI - in combination with CD81 and CLDN1 - is required to allow HCV entry and support the notion that hSR-BI is an essential entry factor of HCV.

We used these complementation assays to characterize the properties of hSR-BI in HCV entry. HDL, the main ligand of SR-BI, enhances infection of HCVpp [25],[28],[29] and HCVcc [26],[27],[30] in SR-BI-positive human cells that are susceptible to HCV entry like, e.g., Huh-7, HepG2-CD81, PLC/PRF/5, SW13 or Hep3B cells. We found that HCVpp entry into both BRL3A-CD81-CLDN1-SR-BI and SK-Hep1-CLDN1-SR-BI cells was stimulated by HDL to levels comparable to those detected in human hepatoma cells (Figure 1C). No change of cell surface (co)-expression of hSR-BI and/or hCD81 could be detected upon incubation of these cells with HDL (Figure S2 and data not shown).

**Figure 2:**
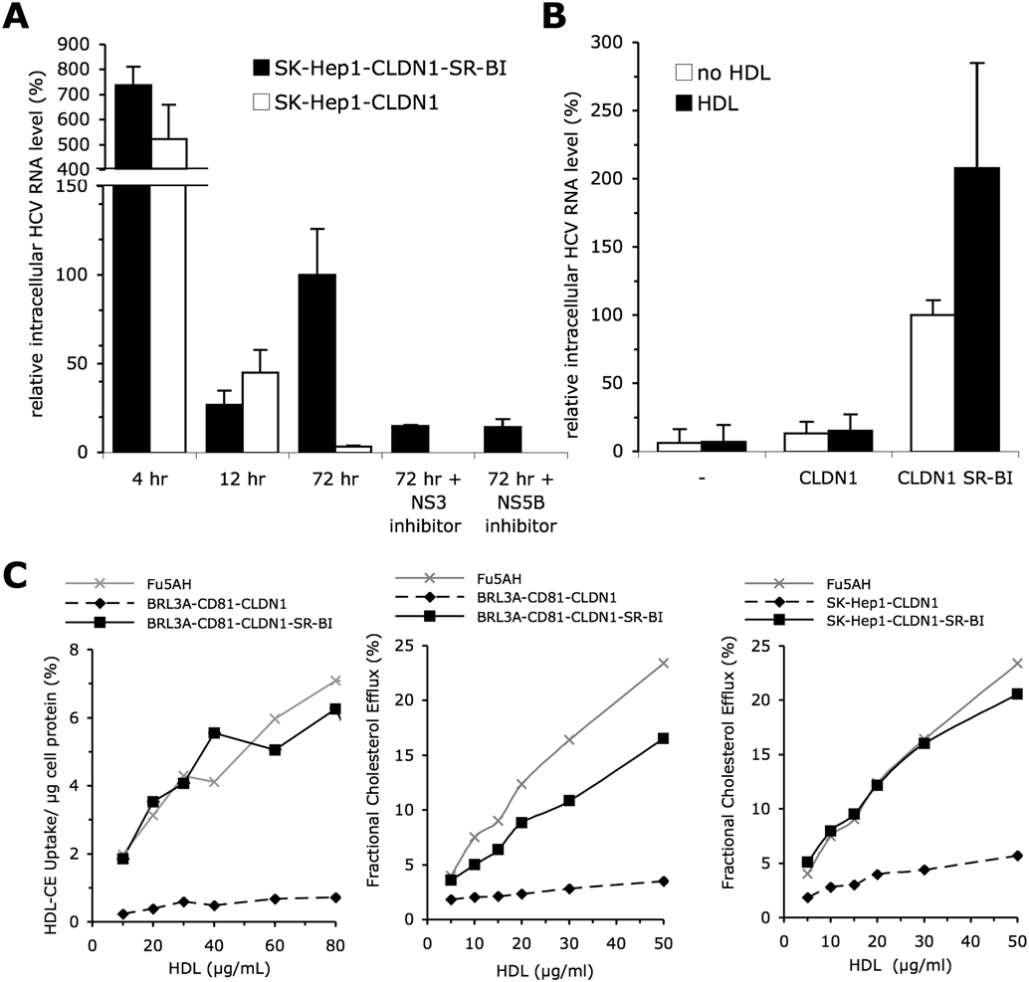
HCVcc entry upon ectopic hSR-BI co-expression with other HCV receptors. (A) Results of HCVcc entry assays, assessed by measuring intracellular HCV RNA level at 4, 12, and 72 hr post-infection, in SK-Hep1 cells ectopically expressing CLDN1 vs. CLDN1 and SR-BI, using cell culture–produced HCVcc in the absence or in the presence of NS3 protease inhibitor and NS5B-dependent RNA synthesis inhibitor (BILN2051 and 2′-C-methyl-adenosine, respectively, kindly provided by FV Chisari). Results are standardized with respect to the HCV RNA level obtained at 72 hr on non-treated SK-Hep1-CLDN1-SR-BI cells (mean±SD; n = 3). Intracellular HCV RNA levels of SK-Hep1-CLDN1-SR-BI was on average 440-fold lower than HCV RNA levels measured in Huh7.5 cell (4.7×10^3^±2.4×10^2^ genome copies per µg cellular mRNA) using the same HCVcc supernatants. (B) Results of HCVcc entry assays, assessed at 72 hr post-infection, in SK-Hep1 cells ectopically expressing the indicated HCV entry factors, using cell culture–produced HCVcc in the absence (white bars) or presence (black bars) of 0.6 µg/ml cholesterol-HDL. Results are standardized with respect to HCV RNA level obtained in the absence of HDL on wt SR-BI–expressing SK-Hep1-CLDN1 cells (mean±SD; n = 5). Similar experiments in BRL3A-CD81-CLDN1-SR-BI cells allowed detection of HCV RNA at 72 hr, which was specifically increased upon treatment with HDL, and revealed differences upon expression of HCV entry factors (data not shown) consistent with the results obtained with HCVpp. However, kinetic experiments and use of replication inhibitors did not allow firm demonstration of HCV replication in these cells. (C) Dose-response curves for the SR-BI–dependent free cholesterol efflux and for HDL-CE uptake determined in BRL3A-CD81-CLDN1 and SKHep1-CLDN1 cells ectopically expressing, or not, hSR-BI, or in Fu5AH cells expressing high endogenous levels of rat SR-BI. Cholesterol efflux is expressed as the percentage of total labelled ^3^H-cholesterol released to the medium. Selective HDL-CE uptake is expressed as the percentage of labelled HDL-CE delivered to cells per µg of cell protein. The values represent the means ±SD of three experiments.

Next, we confirmed the above findings using cell culture-derived HCV (HCVcc). In comparison to HCVcc infection of Huh-7.5 cells, for which viral infectious titers (above 1×10^6^ i.u./ml) could be assessed by immunostaining for Core protein, the same viral stocks resulted in lower infectivity levels in SK-Hep1-CLDN1-SR-BI target cells (data not shown), precluding accurate determination of infectious titers by immuno-detection. Therefore, we used a sensitive and quantitative real-time RT-PCR (RT-qPCR) assay to measure changes in HCV RNA at 4 hr, 12 hr and 72 hr post-infection with HCVcc. Inoculation of HCVcc on SK-Hep1-CLDN1 and SK-Hep1-CLDN1-SR-BI cells yielded strong RT-qPCR signals at 4 hr post-infection, indicating binding and/or capture of viral particles [9], that progressively declined with 10–30 fold loss at 12 hr (Figure 2A). Yet, while HCV RNA decreased again by ca. 15-fold in the parental SK-Hep1-CLDN1 cells at 72 hr, the HCV RNA levels increased in SK-Hep1-CLDN1-SR-BI cells at 72 hr vs. 12 hr post-infection but were strongly reduced when such target cells were treated during 72 hr with HCV replication inhibitors (BILN2061, an NS3 protease inhibitor, or 2′-C-methyl-adenosine, an NS5B polymerase inhibitor) (Figure 2A). Altogether, these results indicated that the detection of HCV RNAs revealed true HCVcc entry of these cells, leading to viral replication, rather than just residual cell attachment of viral particles. Overall, the co-expression of SR-BI and CLDN1 in SK-Hep1 cells allowed ca. 10 to 20 fold increased HCV RNA detection at this 72 hr time point as compared to SK-Hep1 cells expressing CLDN1 alone or none of these entry factors (Figure 2B), consistent with results obtained with HCVpp (Figure 1B and Figure S1D). Importantly, addition of HDL during infection could stimulate HCV RNA detection in SR-BI-expressing SK-Hep1-CLDN1 cells but not in the parental cells (Figure 2B).

In previous studies [25],[29],[35], it was proposed that one of the mechanisms by which HDL enhances infection could involve SR-BI physiological activity. To address this possibility, we first sought to demonstrate whether SR-BI expressed in the BRL3A and SK-Hep1 cellular backgrounds was functional and could mediate lipid transfer, i.e., selective uptake of ^3^H-CE-labelled HDL [36] and efflux of ^3^H-cholesterol to phospholipid cholesterol acceptors [37]. As shown in Figure 2C, while BRL3A-CD81-CLDN1 cells could not or hardly mediate lipid transfer most likely owing to undetectable SR-BI levels (Figure S1A and S1B), ectopic expression of hSR-BI in these cells resulted in efficient lipid uptake and efflux, as compared to rat hepatoma Fu5AH cells expressing rat SR-BI (Figure S1A and S1B) used as positive controls. Likewise, over-expression of hSR-BI in SK-Hep1-CLDN1 cells resulted in efficient cholesterol efflux as compared to parental cells (Figure 2C). Importantly, these results were consistent with the restoration of HDL-mediated enhancement of HCV entry upon ectopic expression of SR-BI (Figure 1C and Figure 2B).

Altogether, these results established that BRL3A-CD81-CLDN1 and SK-Hep1-CLDN1 cells provide original and useful tools for SR-BI complementation assays and render for the first time mutagenetic approaches possible to study the roles of SR-BI in HCV entry.

### Function of SR-BI intracellular domain

**Figure 3:**
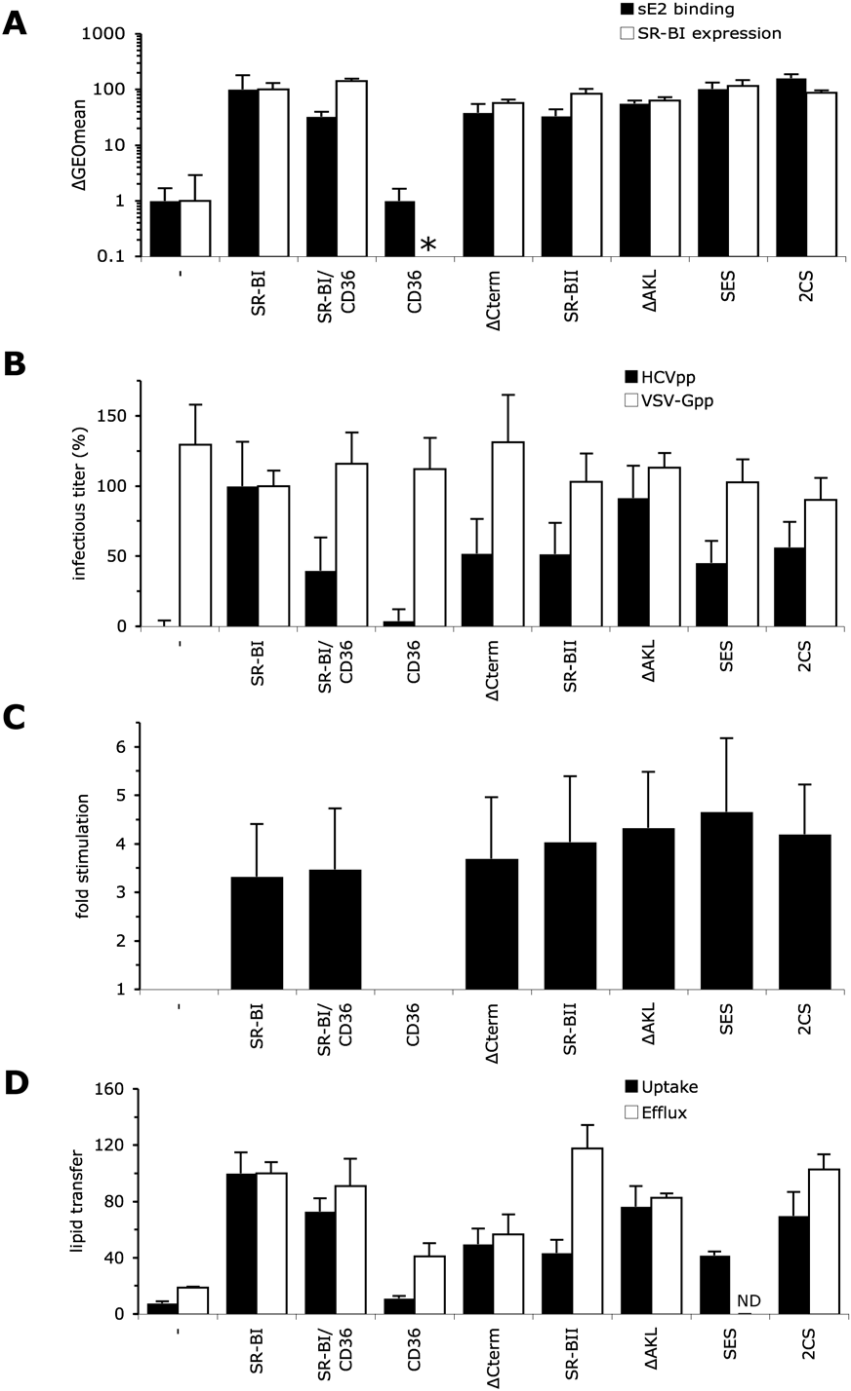
HCVpp entry in BRL3A-CD81-CLDN1 cells expressing SR-BI intracellular domain mutants. (A) Cell surface expression (white bars) and sE2 binding (black bars) of SR-BI mutants/isoforms was determined using anti-SR-BI antibody (CLA-1 mAb) and soluble E2 protein (sE2), respectively. The results of cell surface expression, analyzed by flow cytometry of BRL3A-CD81-CLDN1 cells transduced with retroviral vectors carrying the indicated SR-BI mutants, are expressed as the average percentages of GEOmean (geometric mean) fluorescence shifts (mean±SD; n = 3) detected between mutant receptor–expressing cells and parental (—) cells, relative to cells expressing wild-type SR-BI (ca. 20-fold of GEOmean shift, see Figure 1A) set to 100. The results of sE2 binding are expressed as the average percentages of GEOmean fluorescence shifts (mean±SD; n = 3) detected in parental BRL3A cells (—) or in BRL3A cells only expressing the indicated SR-BI mutants that were incubated with sE2-containing medium vs. sE2-free medium, relative to cells expressing wild-type SR-BI (ca. 10-fold GEOmean shift, see Figure 6) set to 100. Cell surface expression of SR-BI mutants in these latter cells was similar to that detected in the SR-BI–expressing BRL3A-CD81-CLDN1 cells (data not shown). Cell surface expression of CD36 (*, data not shown) was verified using a CD36 antibody (FA6-152, abcam). (B) Effect of SR-BI mutations on infectivity of HCVpp produced in serum-free media. The results of infectivity (mean±SD; n = 5) are expressed relative to the infectious titers of HCVpp or of control VSV-Gpp particles determined on wt SR-BI–expressing BRL3A-CD81-CLDN1 cells (input ca. 10^4^ GFP IU) set to 100. (C) Results of HCVpp infection-enhancement induced by HDL (6 µg/ml cholesterol-HDL), expressed as ratios between average infectious titers determined in the presence or absence of HDL (mean±SD; n = 5). No changes of infectivity of VSV-Gpp control particles were detected under these experimental conditions (data not shown), as reported previously [25]. (D) Relative capacities of SR-BI mutants to mediate HDL-CE uptake (black bars) and free cholesterol efflux (white bars) relative to wt SR-BI set to 100 (mean±SD; n = 3). ND, not determined.

We tested a panel of well-characterized SR-BI mutants in receptor complementation assays in order to address the properties of its intracellular and extracellular domains. Upon introduction in BRL3A-CD81-CLDN1 or SK-Hep1-CLDN1 cells, each SR-BI mutant was studied for its capacity to mediate HCV-E2 binding, HCV entry and HDL-induced infection-enhancement. Because some mutations introduced into human SR-BI were originally characterized in the murine ortholog, which shares 79% identity with human SR-BI (data not shown), we also characterized lipid uptake and efflux mediated by these human SR-BI mutants. To address the functions of the SR-BI intracellular domain, we expressed several SR-BI mutants or isoforms in BRL3A-CD81-CLDN1 and SK-Hep1-CLDN1 cells. By adjusting the input of vectors used to transduce the mutant/chimeric receptors in these cells, we obtained cell surface expression levels comparable to that of wt SR-BI as detected by FACS analysis (Figure 3A). To investigate the capacity of these modified SR-BI receptors to mediate binding of HCV surface glycoproteins (Figure 3A), we used a soluble recombinant form of HCV E2 glycoprotein (sE2), which harbors determinants of binding to CD81 [38],[39] and to SR-BI [7]. Of note, sE2-based binding assays may not fully represent all the binding parameters of HCV particles to CD81 [9],[40],[41],[42]. Yet, we decided to use this assay since in previous studies [27],[35],[43], we found that SR-BI-binding of HCVpp parallels that of sE2 although the latter is more sensitive than binding of HCVpp. Importantly, all these different target cells induced comparable entry levels of control viral particles harboring the VSV-G glycoprotein (VSV-Gpp), with less than 30% variation of VSV-Gpp titers as compared to those detected on wt SR-BI-expressing cells (Figure 3B). Note that although Figure 3B provides the raw data, the small differences of VSV-Gpp titers between the different SR-BI mutant-expressing cell lines could be used to normalize HCVpp infectivity.

First, we investigated the HCV entry properties of a chimeric SR-BI/CD36 receptor [44],[45],[46], in which the two transmembrane domains and cytoplasmic tails of SR-BI were replaced by those of CD36, a close homolog of SR-BI that mediates high-affinity HDL binding but not efficient lipid transfer [46]. Cells expressing wt CD36 did not induce sE2 binding (Figure 3A), as reported previously [7], nor did they allow HCVpp entry (Figure 3B). In contrast, as compared to wt SR-BI, the SR-BI/CD36 chimera mediated about 3-fold reduced sE2 binding, in agreement with the 3-fold reduction of HCVpp entry, after VSV-Gpp normalization. Despite this reduced basal HCV entry, this mutant receptor allowed HDL-mediated infection-enhancement at levels similar to those obtained with wt SR-BI (Figure 3C).

To examine further the involvement of the SR-BI endodomain in HCV entry, we expressed in BRL3A-CD81-CLDN1 cells SR-BI forms harboring alterations of its C-terminal cytoplasmic tail, which contains signals associated to SR-BI expression, localization and/or function: ΔCterm, a SR-BI mutant lacking the C-terminal cytoplasmic tail [45], and SR-BII, an alternative mRNA splice variant of SR-BI with an entirely different cytoplasmic C terminus that promotes more rapid HDL/SR-BII endocytosis as compared to SR-BI and alternative signaling events [47],[48]. Both forms of SR-BI were previously shown to mediate efficient binding of HDL [45],[47] and to induce lipid transfer (Figure 3D). We adjusted SR-BII cell surface expression to levels similar to those of SR-BI, but albeit all our efforts the expression levels of the ΔCterm mutant remained two-fold reduced (Figure 3A). Similar to results obtained with the SR-BI/CD36 chimera, these altered SR-BI receptors induced reduced sE2 binding (by 2–3 fold) and HCVpp entry (by 2-fold) but wild type level of HDL-induced infection-enhancement (Figure 3A–3C). Altogether, the results obtained with SR-BII, SR-BI/CD36 or ΔCterm mutants indicated that while the cytoplasmic tail of SR-BI does not seem to be involved in stimulation of infection by HDL, it could influence the basal HCV entry efficiency. Furthermore, the results on sE2 binding suggested that the level of cell attachment of viral particles was altered for these mutants, which could reflect alterations of receptor affinity, density, localization and/or turnover at the plasma membrane.

We therefore sought to investigate specific determinants of SR-BI cytoplasmic tail that could modulate HCV entry. We first generated a mutant SR-BI receptor, ΔAKL, in which we removed a carboxy-terminal motif of SR-BI that mediates interaction with PDZKI or CLAMP [49]. PDZKI is a four-PDZ-domain-containing protein that is associated with SR-BI in hepatocytes and that may stabilize SR-BI in the sinusoidal plasma membrane by modulating its intracellular transport, localization, assembly and scaffolding [49],[50]. Compared to wt SR-BI, we found that the deletion of PDZKI-associating motif slightly reduced mutant receptor expression and sE2 binding by less than 2-fold but had no or minor influence on HCV entry and HDL-mediated infection enhancement (Figure 3). Furthermore, when PDZK1 was knocked-down in Huh-7 cells using siRNAs, we found that the PDZK1 down-regulated cells induced HCV entry and infection-enhancement at levels identical to those detected in unmodified Huh-7 cells (Figure S3). Thus, PDZK1 down-regulation had almost no effect on HCV entry, in agreement with the results of AKL motif deletion. Next, we generated alternative cytoplasmic-tail mutants: SES, in which the endocytosis motif of SR-BII was functionally introduced [51], and 2CS, in which the two acylation sites of the SR-BI carboxy-terminal cytoplasmic tail were mutated [46]. Like the ΔAKL mutant, these alternative mutants allow efficient HDL binding [46],[51]. Despite similar cell surface expression and sE2 binding, as compared to wt SR-BI (Figure 3A), the SES and 2CS mutants induced about 2-fold reduced HCV entry (Figure 3B), hence suggesting a role of the endocytic trafficking and/or membrane localization of SR-BI in HCV entry.

**Figure 4:**
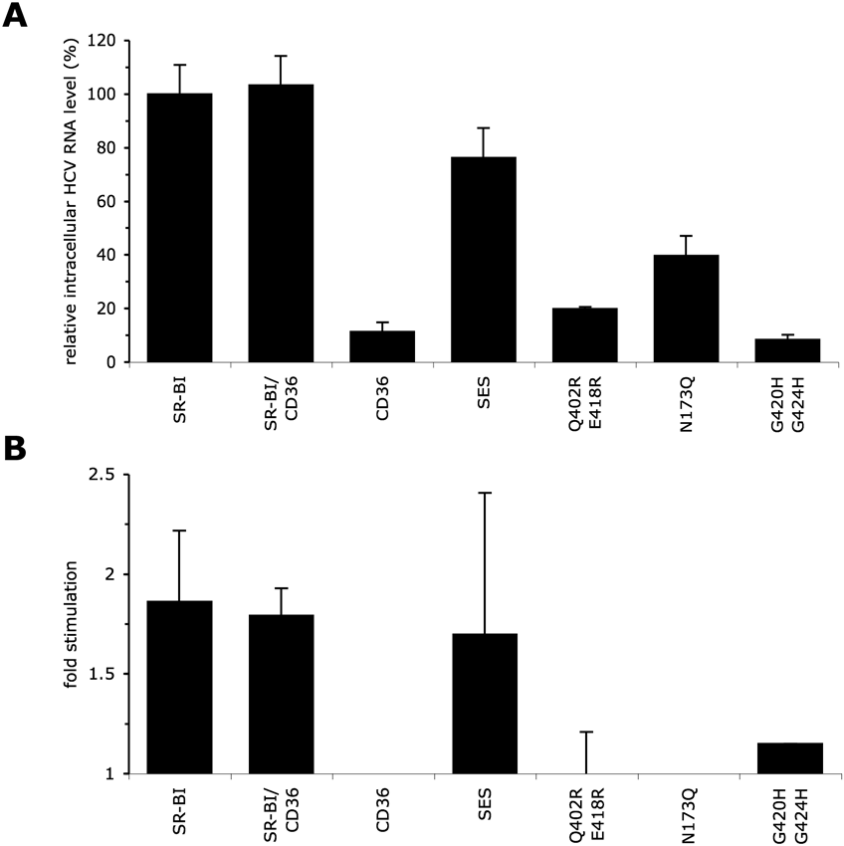
HCVcc entry in SK-Hep1-CLDN1 cells expressing SR-BI mutants. The SR-BI mutants tested were the SR-BI/CD36 chimera and the SES mutants, altering SR-BI intracellular domain, the Q402R/E418R, N173Q, and G420H-G424H mutants, altering SR-BI ectodomain. (A) Effect of SR-BI mutations on infectivity of HCVcc produced under standard conditions and assessed by measuring intracellular HCV RNA level at 72 hr post-infection (see Figure 2A). The results of infectivity (mean±SD; n = 3) are expressed relative to HCVcc infection of wt SR-BI–expressing SK-Hep1-CLDN1 cells, which was set to 100. (B) Results of HCVcc infection-enhancement induced by HDL (0.6 µg/ml cholesterol-HDL) on wt SR-BI–expressing SK-Hep1-CLDN1 cells, expressed as ratios relative to infection in the absence of HDL of the same cells set to 100 (mean±SD; n = 3). Similar experiments in BRL3A-CD81-CLDN1 cells expressing these SR-BI mutants were performed (data not shown) and were consistent with the results obtained in SK-Hep1-CLDN1 cells.

While these results were obtained in a rat hepato-carcinoma background, they were largely confirmed in the human background of SK-Hep1-CLDN1 cells expressing the different SR-BI mutants (Figure S4). Furthermore, we tested a subset of these SR-BI mutants in HCVcc infection assays. While CD36 expression in SK-Hep1-CLDN1 cells did not induce infection of HCVcc, the SR-BI/CD36 chimera and the SES mutant were functional, but, for the latter, infection was reduced in comparison to wt SR-BI (Figure 4), in line with results obtained with HCVpp infection assays. Finally, we found that HDL stimulated HCVpp entry (Figure 3 and Figure S4) and HCVcc (Figure 4) at similar levels for SR-BI cytoplasmic tail mutants as compared to wt SR-BI. Altogether, these results indicated that the C-terminal cytoplasmic tail of SR-BI modulates the basal HCV entry process, but seems not to influence HDL-mediated infection-enhancement. This latter observation is consistent with the fact that the SR-BI C-terminal mutants mediated efficient lipid transfer (Figure 3D).

### Functions of the extracellular domain

**Figure 5:**
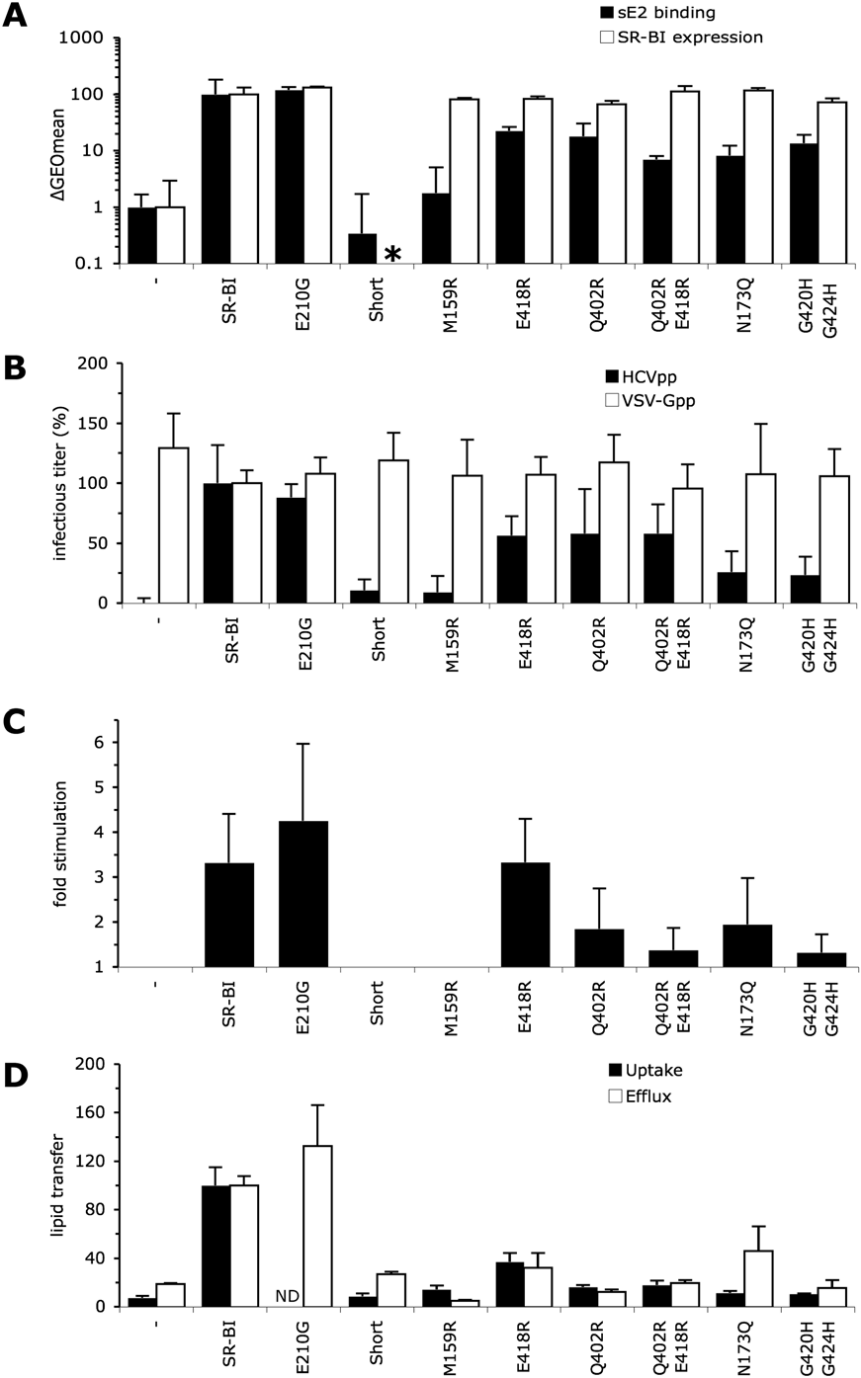
HCVpp entry in BRL3A-CD81-CLDN1 cells expressing SR-BI ectodomain mutants. (A) Cell surface expression (white bars) and sE2 binding (black bars) of SR-BI mutants/isoforms as determined using anti-SR-BI antibody (CLA-1 mAb) and soluble E2 protein (sE2), respectively. The results of cell surface expression, analyzed by flow cytometry of BRL3A-CD81-CLDN1 living cells transduced with retroviral vectors carrying the indicated SR-BI mutants, are expressed as the average percentages of GEOmean (geometric mean) fluorescence shifts (mean±SD; n = 3) detected between mutant receptor–expressing cells and parental (—) cells, relative to cells expressing wild-type SR-BI (ca. 20-fold of GEOmean shift, see Figure 1A) set to 100. The results of sE2 binding are expressed as the average percentages of GEOmean fluorescence shifts (mean±SD; n = 3) detected in parental BRL3A cells (—) or in BRL3A cells only expressing the indicated SR-BI mutants that were incubated with sE2-containing medium vs. sE2-free medium, relative to cells expressing wild-type SR-BI (ca. 10-fold of GEOmean shift, see Figure 6) set to 100. Cell surface expression of SR-BI mutants in these latter cells was similar to that detected in the SR-BI-expressing BRL3A-CD81-CLDN1 cells (data not shown). Cell surface expression of SR-BI-Short (*, data not shown) was verified by immuno-blotting using an antibody against SR-BI C-terminus (400-104, Novus) on surface-biotinylated proteins that were purified with streptavidin-coated beads. (B) Effect of SR-BI mutations on infectivity of HCVpp produced in serum-free media. The results of infectivity (mean±SD; n = 5) are expressed relative to the infectious titers of HCVpp or of control VSV-Gpp particles determined on wt SR-BI–expressing BRL3A-CD81-CLDN1 cells (input ca. 10^4^ GFP i.u.), set to 100. (C) Results of HCVpp infection-enhancement induced by HDL (6 µg/ml cholesterol-HDL), expressed as ratios between average infectious titers determined in the presence or absence of HDL (mean±SD; n = 5). No changes of infectivity of VSV-Gpp control particles were detected under these experimental conditions (data not shown), as reported previously [25]. (D) Relative capacities of SR-BI mutants to mediate HDL-CE uptake (black bars) and free cholesterol efflux (white bars) relative to wt SR-BI set to 100 (mean±SD; n = 3). ND, not determined.

To address the functions of the SR-BI ectodomain, we expressed SR-BI mutants that exhibited reduced HDL binding and/or lipid transfer properties. The capacity of either mutant to mediate sE2 binding, HCV entry and HDL-mediated infection-enhancement was compared with wt SR-BI or with the E210G ectodomain mutant, exhibiting wt lipid transfer properties (Figure 5D). First, we expressed a shorter isoform of SR-BI (SR-BI-Short), produced by alternative splicing of SR-BI mRNA that removes 100-amino-acids of the ectodomain [18], and the M159R point mutant, targeting a motif conserved between mouse and human SR-BI that has been shown to reduce HDL binding and lipid transfer [52]. The decreased levels of lipid uptake and efflux were verified for each mutant (Figure 5D). Upon expression in BRL3A-CD81-CLDN1 cells, these modified SR-BI receptors mediated no (SR-BI-Short) or hardly detectable (M159R) sE2 binding (Figure 5A) and reduced HCVpp entry by over 10-fold, in comparison to wt SR-BI (Figure 5B). These results therefore highlighted the importance of E2 attachment to the SR-BI ectodomain in the HCV entry process. Moreover, these mutants were unable to induce infection-enhancement when HDL was added during HCVpp infection (Figure 5C), which could be due to their inability to mediate HCV binding or, alternatively, from their inability to bind HDL and/or to mediate lipid transfer.

**Figure 6:**
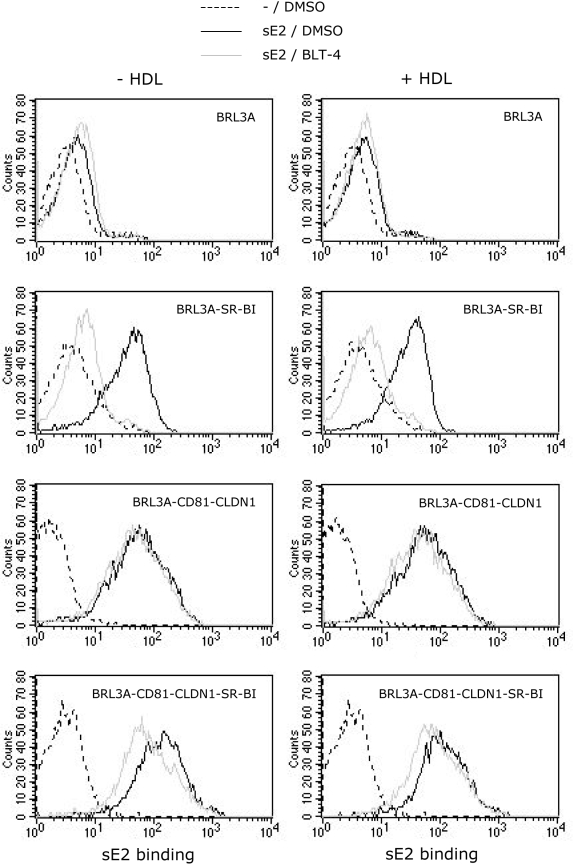
Inhibition of sE2 binding to SR-BI by BLT-4 compound. BRL3A cells expressing the indicated entry factors were incubated for 1 hr at 37°C with soluble E2 protein (sE2) (plain lines) or without sE2 (dotted lines) at saturating concentrations, after pre-incubation for 45 min at 37°C with (gray lines) or without (black lines) 50 µM BLT-4 (Chembridge), a SR-BI inhibitor [53], and with (left panels) or without (right panels) HDL (6 µg/ml cholesterol-HDL). SR-BI expression was not modified by the incubation with BLT-4 (data not shown). The data are representative of three independent experiments.

BLTs (block lipid transfer; BLT-1 to BLT-4) are small lipid transport inhibitors originally identified in a high-throughput chemical screen of intact mSR-BI-expressing cells [53]. They inhibit SR-BI-dependent selective cholesterol uptake and efflux from and to HDL, but do not block HDL binding. We, and others, previously showed that BLTs also inhibit HDL-mediated HCV infection-enhancement, which, together with alternative results using SR-BI blocking antibodies or SR-BI down-regulation, suggested the possibility that the SR-BI physiological activity is involved during HCV entry [25],[29]. Surprisingly, here we found that BLT-4, characterized in several previous studies [25],[29], inhibited sE2 binding to SR-BI-expressing BRL3A (Figure 6) and CHO (data not shown) cells, at the same concentrations as those that proved to be effective for HCV entry inhibition, i.e., 15–50 µM [25],[27],[29]. Similar inhibition of HCVpp infectivity and sE2 binding was detected with other BLTs (data not shown). While sE2 readily bound BRL-CD81-CLDN1 cells, no inhibition of sE2 binding by BLTs could be detected on those cells (Figure 6 and data not shown). Moreover, while co-expression of CD81 and SR-BI increased sE2 binding as compared to BRL3A cells expressing either entry factor, BLT-4 reduced sE2 binding to BRL-CD81-CLDN1-SR-BI at the levels detected on BRL-CD81-CLDN1 cells (Figure 6). These results indicated that BLTs specifically inhibited sE2 binding to SR-BI. Moreover, HDL did not increase sE2 binding to either SR-BI or CD81 and did not modify inhibition of sE2 binding to SR-BI by BLT-4 (Figure 6). The unexpected finding that BLTs inhibit sE2/SR-BI binding further lent support for a requirement of sE2 binding to SR-BI for HCV entry. However, since the use of BLTs could not unambiguously demonstrate that HDL-mediated infection-enhancement requires SR-BI-dependent lipid transfer, next, we expressed and analyzed effects of SR-BI ectodomain mutants E418R, Q402R and Q402R/E418R that have reduced lipid uptake properties [52],[54], as shown in Figure 5D. Similar levels of cell surface expression for either mutant could be detected by FACS analysis, as compared to wt SR-BI (Figure 5A). These SR-BI mutants exhibited 5–15 fold reduced sE2 binding (Figure 5A), which was correlated with a lower infectivity of HCVpp (Figure 5B), in agreement with results obtained with the M159R and SR-BI-Short mutants. When HDL was added during the HCV entry assay, we found that the extent of infection-enhancement (Figure 5C) was correlated to the capacity of these SR-BI mutants to mediate lipid uptake (Figure 5D). For example, while E418R that shows 60% reduced lipid uptake capacity induced wild-type HDL-mediated infection-enhancement, the most disabled lipid transfer mutants, Q402R and Q402R-E418R, had lost almost all infection-enhancement capacity (Figure 5C and 5D). Collectively, the results demonstrated that SR-BI functions as an HCV entry factor by providing both cell surface binding sites and lipid uptake activity.

Importantly, these different mutants had reduced HDL binding levels [52],[54], which could not unambiguously discriminate between altered HDL binding vs. reduced lipid transfer, the reason for loss of HDL-mediated infection-enhancement induced by SR-BI. Therefore, we next generated the N173Q and G420H-G424H mutants that have impaired lipid transfer but normal HDL binding [55],[56],[57]. These mutants induced ca. 10-fold reduced sE2 binding (Figure 5A), which resulted in ca. 4–5 fold reduced infectivity (Figure 5B). Interestingly, while the N173Q mutant poorly induced HDL-mediated infection-enhancement, the G420H-G424H failed to support stimulation of infection induced by HDL (Figure 5C), in agreement with their strongly reduced capacity to mediate lipid transfer (Figure 5D). These data further supported the notion that the lipid transfer functions of SR-BI are required for HCV entry enhancement.

These results, obtained in the background of BRL3A rat cells, were confirmed in the human background of SK-Hep1 cells (Figure S4). Furthermore, we performed HCVcc infection assays of SK-Hep1-CLDN1 cells expressing a subset of mutants of SR-BI extracellular domain (i.e., Q402R-E418R, N173Q and G420H-G424H) (Figure 4). We confirmed that these mutant receptors reduced infection in comparison to wt SR-BI, but completely abolished HDL-mediated infection enhancement.

## Discussion

Several lines of evidence suggested that SR-BI plays a prominent role in HCV entry into cells. First, SR-BI provides both direct and ApoB-mediated interaction with HCV particles [7],[24],[58]. Second, antibody blocking or down-regulation of SR-BI inhibit HCV entry in permissive cells [12],[14],[25],[26],[29],[59]. Third, HDL, the major important ligand of SR-BI, stimulates HCV entry in human hepatocarcinoma target cells [25],[26],[27],[28],[29],[35]. Finally, using small molecules that inhibit the selective lipid transfer functions of SR-BI, named BLTs [53], it was proposed that besides providing a docking site for HCV particles, SR-BI physiological property, i.e., cholesterol uptake and/or efflux, could be exploited during HCV entry [25],[29]. Yet, despite this indirect evidence, functional complementation assays addressing the implication of SR-BI during HCV entry were lacking since no cell type in which SR-BI ectopic expression would restore HCV infection has been available.

Aiming to directly address these different possible functions of SR-BI in HCV entry, we report here for the first time a sensitive functional complementation assay that allows studying this molecule by mutagenesis. By screening cells of human and non-human origins for absence or low SR-BI expression in which HCV entry could be restored by SR-BI ectopic expression, our data highlight one non-hepatoma human cell line and one hepatoma rat cell line in which HCV entry assays could be performed. Our results therefore clearly demonstrate that SR-BI is an essential entry factor, along with CD81 and CLDN1, mediating HCV entry. This is also the first report showing that HCV can enter non-human cells upon expression of HCV receptors. Importantly, we show that the selective lipid transfer properties of human SR-BI were fully functional in such heterologous cell backgrounds, allowing us to directly address the role of specific residues of SR-BI ecto- and endo-domains in HDL-mediated infection-enhancement.

How HCV undergoes cell penetration following binding to its specific receptors remains ill defined. SR-BI alone or, alternatively, SR-BI interacting with the other HCV receptors may initiate cell penetration and/or mediate virus internalization. That HCVcc binds SR-BI-expressing CHO cells but not CD81-expressing CHO cells [9] may imply that a first contact with SR-BI is necessary before the viral particle can interact with CD81. SR-BI may induce HCV endocytosis by itself, as suggested by its capacity to mediate internalization of its natural ligands [21],[60],[61]. Interestingly, expression of SR-BII, a mRNA splice variant that differs from SR-BI at the C-terminus [18], which confers intracellular localization of SR-BII and rapid internalization of HDL [19],[47], reduces HCVpp entry as compared to SR-BI (this report). Along with findings of others, i.e., *via* SR-BII over-expression in Huh-7 cells [59], this indicates that SR-BI, rather than SR-BII, is a preferred receptor for HCV entry and that determinants of SR-BI cytoplasmic tail different from those controlling its endocytosis may regulate HCV entry. Additionally, deletion (ΔCterm mutant) or replacement (SR-BI/CD36 chimera) of SR-BI cytoplasmic tail also reduced its capacity to mediate HCV entry. Moreover, functional restoration of the SR-BII dileucine endocytic motif in the SR-BI C-terminal cytoplasmic tail (SES mutant), which induces rapid internalization of SR-BI/HDL complexes [51], did not increase HCV entry but rather reduced it. Altogether, these results suggested that if SR-BI initiates and/or promotes HCV endocytosis, it could be through its interaction with other HCV entry factors rather than *via* a classical binary virus/receptor complex.

In agreement with this assumption, our data raise the possibility that determinants of the C-terminal cytoplasmic tail contribute to SR-BI HCV entry functions through modulation of its intracellular trafficking and/or membrane localization. The study of the ΔAKL mutant, which abrogates SR-BI interaction with PDZK1 that modulates its intracellular transport, localization, assembly and scaffolding [49],[50], did not affect HCV entry, consistent with the lack of effect of PDZK1 down-regulation in Huh-7 cells. However, as suggested by results with the 2CS mutant (C462S-C470S), which prevents SR-BI palmitoylation [46] and thus its potential association to lipid raft micro-domains, our results indicate that localization of SR-BI in specific micro-environments could play a role in HCV entry. Indeed, sub-cellular fractionation experiments showed that SR-BI localizes in plasma membrane lipid rafts [62] and/or caveolae [63], which may play a critical role in SR-BI-mediated transfer of lipids between HDL and cells [64],[65] and, possibly, HCV entry. Such low-density membrane microdomains are enriched in cholesterol and glycolipids, and have been involved in a number of transport and signaling events that could be important for virus endocytosis and intracellular transport [66].

Our study of SR-BI ectodomain mutants provides the first direct and functional evidence that HCV and HDL binding to SR-BI, and intact lipid transfer properties of SR-BI are required for SR-BI function as HCV entry factor. SR-BI-mediated uptake of HDL CE is a two-step process that requires high-affinity binding of HDL followed by incorporation of CE to the plasma membrane pool and subsequent transfer of the lipid to an inaccessible pool. CE uptake is followed by hydrolysis to free cholesterol by a neutral CE hydrolase. SR-BI-mediated lipid uptake leads to increase of cholesterol content of the target cell membrane [22],[23],[65] and activates distinct signaling pathways [67], which may provide different beneficial roles for HCV entry.

First, using liposome-based *in vitro* fusion assays, HCVpp membrane fusion was shown to be facilitated when cholesterol is present in the target membrane [15]. By analogy with fusion processes of Flaviviruses and Alphaviruses that have been widely studied [68], cholesterol-enrichment of target membranes may induce specific curvature that could positively influence the early interactions of HCV fusion protein. Alternatively, local cholesterol enrichment may facilitate binding [69] and/or conformational changes [70] within the HCV glycoproteins that are required for membrane fusion processes.

Second, HCVpp internalization was shown to be specifically accelerated by HDL [27]. As discussed above, this effect is likely to be indirect. Indeed, the internalization rate of HCVpp is significantly slower than that of pseudo-particles harboring the surface glycoproteins from murine leukemia virus, influenza virus [27], Semliki forest virus, or vesicular stomatitis virus [11], and has an half-life much longer than that of HDL internalization [71]. Furthermore, HDL added during the initial stage of infection suppresses an one-hour time lag during which cell-bound virions are not internalized [27]. This may reflect the time interval required to assemble a functional HCV receptor complex, which may be reduced upon SR-BI activation through modifications of the cell membrane. A possibility is that HDL/SR-BI interaction augments the rate of CD81 recruitment at virion-binding sites and/or internalization of HCV/CD81 complexes *via* a cholesterol-dependent pathway. In agreement with this assumption, that SR-BI mutants increasing internalization (SR-BII and SES mutant) are less effective than wt SR-BI to mediate HCV entry (see above) suggests that HCV internalization is likely driven by SR-BI interacting with other receptors rather than *via* SR-BI alone. In this respect, it is interesting that CD81 and SR-BI function cooperatively to initiate HCV infection [32],[72], that CD81-mediated HCV entry seems dependent on membrane cholesterol [72], and that SR-BI/HDL-mediated HCV entry enhancement still requires CD81 [25],[27]. Interestingly, both SR-BI and CD81 have been proposed as cell factors allowing *Plasmodium* sporozoite invasion and/or intracellular parasite development in mouse [73] and human [74] hepatocytes, perhaps through SR-BI-induced regulation of the organization of CD81 at the plasma membrane by mediating an arrangement permissive to penetration by sporozoites [73]. Yet, although SR-BI lipid transfer blockers, i.e., SR-BI antibodies and BLTs, reduce both *Plasmodium* and HCV infection [25],[29],[74], there seems to be important differences in the mechanisms involved. Indeed, in contrast to HCV surface glycoproteins, *Plasmodium* sporozoites do not seem to directly interact with SR-BI and/or CD81 [73],[75]. Furthermore, in contrast to HCV, *Plasmodium* sporozoites invasion is not enhanced by HDL [74]. Finally, while we demonstrated that cholesterol uptake mediated by SR-BI ectopically expressed in BRL3A-CD81-CLN1 cells is functional (Figure 2C), we did not notice up-regulation of CD81 cell surface expression and TEM localization whether these cells expressed or not SR-BI and following treatment with HDL (Figure S2 and data not shown), in contrast to other studies that revealed such CD81 changes in mouse hepatocytes [73]. These differences may reflect dissimilar properties of mouse vs. human SR-BI and CD81, of species-specific cholesterol transport processes and/or of the cellular backgrounds used [76]. Likewise, no CD81 changes could be detected in Huh-7 cells incubated in the presence or in the absence of HDL (Figure S2). Consistently, HDL did not enhance sE2 binding capacity to SR-BI and/or CD81 (Figure 6). Finally, recent results of others suggest that the association of CD81 with TEM is not essential for HCV entry (Dr Jean Dubuisson, Personal Communication). Alternatively, homo-oligomerization of SR-BI seems associated with functional expression of the selective HDL cholesteryl ester uptake pathway [77] and may contribute to the formation of a HCV receptor complex.

Third, an essential component of HDL that seems responsible for infection enhancement at the level of HCV membrane fusion is the apolipoprotein C-I (ApoC-I) [28],[35], an exchangeable apolipoprotein that could be transferred from HDL to the HCV membrane during SR-BI-mediated lipid transfer and could predispose HCV envelope for fusion with a target membrane, *via* alterations of its outer phospholipid layer [35].

Further analysis of the HCV entry events mediated by HCV receptors and co-factors will be greatly facilitated by the availability of the novel functional receptor-complementation assay described in this report. Moreover, it opens the way to develop small animal models susceptible for HCV in which entry inhibitors can be tested *in vivo*.

## Materials and Methods

### Cell lines

Huh-7 [78], PLC/PRF/5 human hepatoma (ATCC CRL-8024), Hep3B human hepatocellular carcinoma (ATCC HB-8064), BRL3A rat hepatocytes (ATCC CRL-1442) and 293T (ATCC CRL-1573) cells were grown in DMEM (Invitrogen) supplemented with 10% fetal bovine serum (FBS) (Invitrogen). Fu5AH rat hepatoma cells [79] were grown in Eagle's MEM supplemented with 1% L-glutamine and 5% newborn calf serum. CHO (ATCC CRL-1582) and SK-Hep1 (ATCC HTB-52) cells were maintained in RPMI (Invitrogen) with 10% FBS.

### Expression constructs and establishment of cells lines expressing CLDN1, CD81, and SR-BI wt/mutants

Retroviral vectors expressing human CD81 (GenBank accession number: NM_004356), Claudin-1 (NM_021101) and SR-BI (Z22555) or mutant SR-BI receptors were inserted in CNC MLV (murine leukemia virus) vector backbones (kind gift of M. Collins) harboring selectable marker genes for blasticidin, neomycin and hygromycin respectively. Construct details are available upon request. The CD36 was kindly provided by Brian Seeds [80]. The cDNAs encoding the SR-BI-Short [18] and SR-BII [47],[48] were based on the original sequence of human SR-BI [18] and were inserted in the CNC expression vector. The SRBI-CD36 [44],[45],[46], the ΔCterm [45], ΔAKL [49], SES [51] chimeras were previously described for rodent SR-BI and were used to derive the equivalent human SR-BI chimeras investigated in this work. Point mutants encoding the following SR-BI receptors: M159R, N173G, Q402R, E418R, Q402R-E418R, G420H-G424H, C462S-C470S (2CS) [46],[52],[54],[55],[56],[57] and E210G (MD and FLC, unpublished data), were introduced in human SR-BI cDNA by site directed mutagenesis (primer sequences are available upon request). All mutants were sequenced to ensure that the clones possessed only the expected mutation. Retroviral vectors containing CD81, CLDN1 and wt or mutant SR-BI receptors were produced from 293T cells as VSV-G-pseudotyped particles as described previously [81],[82]. Stable expression of either receptor in target cells was obtained by transduction with vector particle-containing supernatants of 293T producer cells, followed by antibiotic selection.

### Production of HCVpp and HCV entry assays

The expression vector for the E1E2 glycoproteins of HCV strain H77 (AF009606) was described previously [33]. Viral pseudo-particles named HCVpp and VSV-Gpp harboured the glycoproteins of HCV and VSV, respectively, and were produced as described previously [33] by transfection in 293T cells of vectors encoding viral glycoproteins, packaging proteins, and GFP-transfer vector. Prior to harvest viral particles-containing supernatants, producer cells were incubated in DMEM containing 0.1% FCS for 24 hrs.

For infection assays, target cells were seeded 24 hr prior to inoculation. 2 hr prior to infection, target cells were pre-incubated in DMEM containing 0.1% FCS. Then medium was removed and dilutions of viral supernatants were added to the cells and incubated for 4 hr. Where indicated, HDL (Calbiochem) was added to the infection reactions at 6 µg/ml of cholesterol. Supernatants were then removed and the infected cells kept in regular medium (DMEM, 10% FCS) for 72 hr before analysis of the percentage of GFP-positive cells by FACS analysis [33]. The infectious titers were expressed as GFP infection units (i.u.) per ml of HCVpp-containing medium. Infections were controlled by using non-enveloped particles, which resulted in background titers between 10^2^ and 10^3^ GFP i.u./ml.

### Production of HCVcc and infection assays

Plasmid pJFH-1 displaying previously described mutations F172C and P173S in core, as well as N534K in E2 [83], was *in vitro* transcribed using the Megascript T7 kit (Ambion). After DNAse treatment, genomic RNA was purified by two acidic phenol/chloroform extractions and pelleted by isopropanol precipitation. Then, RNA was electroporated into Huh-7.5 cells using Gene Pulser II apparatus (Biorad) and cells were cultured under standard conditions. Virus-containing medium was harvested, pooled and added to target cells as described above. Where indicated, HDL (Calbiochem) was added to the infection reactions at 0.6 µg/ml of cholesterol.

Infected cells were collected by trypsinization, and RNA was prepared (RNeasy; QIAGEN), reverse transcribed (iScript cDNA synthesis kit, Biorad) and quantified with HCV specific (5′-CTTCACGCAGAAAGCGTCTA and 5′-CAAGCACCCTATCAGGCAGT) and house-keeping primers targeting RSP11 in SK-Hep1 and Huh-7 cells (5′-GCCGAGACTATCTGCACTAC and 5′-ATGTCCAGCCTCAGAACTTC) or rat GAPDH in BRL3A cells (5′-GTTACCAGGGCTGCCTTCTC and 5′-GGGTTTCCCGTTGATGACC) using the Platinum SYR Green qPCR super mix kit from Invitrogen on an Applied 7000 apparatus.

### Binding and surface staining assays

Binding of soluble E2 glycoprotein, derived from the H77-E2 was performed as previously described [27],[38]. Briefly, 5 µg/ml of sE2 harbouring a His-tag was incubated for 1 hr at 37°C with 10^6^ target cells. The amount of cell-bound sE2 was determined by FACS analysis using 2 µg/ml of an anti-His tag antibody (pentaHis, Qiagen) and using Allophycocyanine (APC)-conjugated anti-mouse antibodies.

The surface expression of hCD81 and hSR-BI was quantified by FACS analysis from 10^6^ live cells using anti-CD81 mAb (clone JS81, Pharmingen) and anti-SR-BI mAb (CLA-1, BD Biosciences), respectively, added to cells for 1 hr in PBFA at 4°C. After washing, the binding of antibody to the cell surface was detected using RPE- or APC-conjugated anti-mouse antibodies.

### Lipid transfer assays

Lipid efflux assays were performed as previously described [37]. After plating, cells were labeled by incubation with ^3^H-cholesterol (1 µCi/ml) for 48 hr. Subsequently cells were incubated for 24 hr in the presence of BSA (0.5%) and newborn calf serum (25%) for Fu5AH or fetal bovine serum (25%) for BRL3a or SK-Hep1, to allow equilibration of the label. After equilibration, cholesterol acceptors (20 µg phospholipid/ml of isolated HDL) were added in serum-free medium and incubated with cells for 4 hr at 37°C. Fractional cholesterol efflux (expressed as percentage) was calculated as the amount of the label recovered in the medium divided by the total label in each well (radioactivity in the medium+radioactivity in the cells) obtained after lipid extraction from cells in a mixture of 3∶2 hexane-isopropanol (3∶2 v/v). The background cholesterol efflux obtained in the absence of cholesterol acceptor was subtracted from the efflux values obtained with the test samples.

Selective HDL-CE (cholesteryl ester) uptake was performed as previously described [36]. Cells were plated in 24-well tissue culture plates (10^6^ cells/well). Two days after plating, cells were washed 3 times with PBS and once with serum-free medium. Cells were subsequently incubated in the presence of ^3^H-CE-labelled HDL (60 µg protein) diluted in serum-free medium at 37°C for 5 hr. At the end of incubation, the medium was removed and cells were washed 4 times with PBS and incubated in the presence of an excess of unlabelled HDL (100 µg protein) for 30 minutes. Cells were then washed 4 times with PBS and solubilized with 200 µl of NaOH 0.2 N for 15 minutes at room temperature with gentle mixing. Protein content (20 µl) from each well was measured using the Bicinchoninic acid protein reagent (Pierce). The radioactive content of 100 µl of each cell lysate was measured by liquid scintillation counting. Selective uptake was calculated from the known specific radioactivity of radiolabelled HDL-CE and is expressed in µg HDL-CE/µg cell protein.

## Supporting Information

Figure S1 HCVpp entry in receptor-complemented BRL3A or SK-Hep1 cells. (A) Western blot analysis of the indicated HCV receptors in lysates of BRL3A, SK-Hep1, and Huh-7 cells ectopically expressing (+) or not expressing (−) the indicated HCV receptors using SR-BI (CLA-1, BD Bioscience), CD81 (JS81, Pharmingen), and CLDN1 (mouse anti-Claudin-1, Interchim) antibodies. The endogenous rat orthologs of these molecules were detected in BRL3A, Fu5AH rat hepatoma cells, and Huh-7 cells (Huh-7*, Fu5AH*, and BRL3A*) by Western blot analysis using cross-reactive antibodies against SR-BI (400-104, Novus), CD81 (EAT-2, Santa Cruz Biotechnology), and CLDN1 (mouse anti-Claudin-1, Interchim) antibodies. The actin staining (mAb AC74, Sigma-Aldrich) was used to ensure equal input of cell lysates. (B) Abundance of rat (white bars) and human (black bars) SR-BI mRNA levels in Fu5AH, BRL3A, BRL3A-CD81-CLDN1, and BRL3A-CD81-CLDN1-SR-BI cells. Total RNA was then extracted, quantified by Real time quantitative PCR, and normalized to rat β-Gus housekeeping gene (Protocol S1). Expression data were corrected for PCR efficiencies of the target and the reference gene, thus making possible analysis of the expression of one gene relative to the others. (C) Results of HCV entry assays on BRL3A-CD81-CLDN1-SR-BI, BRL3A-CD81-CLDN1, and Huh-7 target cells using HCV pseudo-particles carrying a luciferase marker gene and harboring E1E2 glycoproteins derived from the indicated genotypes/subtypes 1a (H77), 1b (Con-1, UKN1B 12.16), 2a (JFH-1, UKN2A 2.4), 2b (UKN2B 2.8), and 3a (UKN3A 1.28), as indicated [15], control viral particles harboring the VSV-G glycoprotein (diluted 1/100) or no glycoprotein (noENV). Results display average infectious titers, expressed as Luciferase unit (RLU) per 10^5^ target cells (mean±SD; n = 3). (D) Results of HCV entry assays on BRL3A (left panel) and SK-Hep1 (right panel) cells ectopically expressing the indicated HCV receptors using HCV pseudo-particles harboring H77-E1E2 glycoproteins (HCVpp), control viral particles harboring the VSV-G glycoprotein (VSV-Gpp), or no glycoprotein (noENVpp). The viral particles, containing a GFP marker gene, were produced in cell culture media devoid of serum lipoproteins. Results are expressed as percentages of the infectious titers (mean±SD; n = 3) determined in BRL3A cells expressing CD81, CLDN1, and SR-BI (titer: 2×10^4^ i.u./ml) and SK-Hep1 expressing CD81, CLDN1, and SR-BI (titer: 5×10^4^ i.u./ml). As indicated, HCVpp entry assays were performed in the absence (−) or in the presence (HDL) of 6 µg/ml cholesterol-HDL. No changes of infectivity with VSV-Gpp control particles were detected under these experimental conditions (data not shown), as reported previously [25].
(0.19 MB TIF)

Figure S2 Cell surface expression of hCD81 and/or hSR-BI in BRL3A cells. BRL3A cells expressing the indicated entry factors were pre-incubated for 2 hrs in low serum-containing medium (0.1%), then incubated for 1 hr at 37°C in the absence (black lines) or in the presence (gray lines) of HDL (6 µg/ml cholesterol-HDL) before staining with JS81 (left panels) or CLA-1 (right panels) antibodies. The background of fluorescence was provided by staining the cells with the secondary antibodies only (dotted lines).
(0.37 MB TIF)

Figure S3 HCV entry in PDZK1 down-regulated target cells. PDZK1 (GenBank accession number: NM_002614) down-regulation was induced upon expression of specific shRNAs (PDZK1-1: 5′-GCTATGGCTTTCACTTAAAT and PDZK1-2: 5′-GAAAGAAGGCCTATGATTA) via the FG12 lentiviral vector [84] introduced in Huh-7 target cells. As controls, Huh-7 cells were transduced with FG12-derived vectors carrying shRNAs for CLDN1 (5′-AAGTGCTTGGAAGACGAT) and CD81 (5′-GATCGATGACCTCTTCTCC) or were left intact (−). (A) Western blot analysis of PDZK1 in lysates of Huh-7 cells in which these shRNAs were expressed (rabbit polyclonal Ab NB 400-1491, Novus Biologicals). The actin staining (mAb AC74, Sigma-Aldrich) was used to assess cell density. (B) Results of HCV entry assays on Huh-7 cells expressing PDZK1 or control shRNAs using HCV pseudo-particles harboring H77-E1E2 glycoproteins (HCVpp), control viral particles harboring the RD114 glycoprotein (RD114pp), or no glycoprotein (noENVpp). The viral particles, containing a CD90 marker gene, were produced in cell culture media devoid of serum lipoproteins. The results of infectivity (mean±SD; n = 3) are expressed relative to the infectious titers of HCVpp or of control RD114pp determined on intact Huh-7 cells, which were determined 72 hr after infection by measuring CD90 reporter gene expression by FACS analysis using an allophycocyanin (APC)-conjugated anti-CD90 mAb (clone 5E10, BD Pharmingen). As indicated, HCVpp entry assays were performed in the absence (−) or in the presence (HDL) of 6 µg/ml cholesterol-HDL.
(0.09 MB TIF)

Figure S4 HCVpp entry in SK-Hep1-CLDN1 cells expressing SR-BI mutants. (A) Cell surface expression (white bars) of SR-BI mutants/isoforms as determined using anti-SR-BI antibody (CLA-1, BD Bioscience). The results of cell surface expression, analyzed by flow cytometry of SK-Hep1-CLDN1 cells transduced with retroviral vectors carrying the indicated SR-BI mutants, are expressed as the average percentages of GEOmean (geometric mean) fluorescence shifts (mean±SD; n = 3) detected between mutant receptor-expressing cells and parental (−) cells, relative to cells expressing wild-type SR-BI (ca. 40-fold GEOmean shift, Figure 1A) set to 100. Cell surface expression of CD36 (*, data not shown) was verified using a CD36 antibody (FA6-152, abcam). Cell surface expression of SR-BI-Short (*, data not shown) was verified by immuno-blotting using an antibody against SR-BI C-terminus (400-104, Novus) on surface-biotinylated proteins that were purified with streptavidin-coated beads. (B) Effect of SR-BI mutations on infectivity of HCVpp produced in serum-free media. The results of infectivity (mean±SD; n = 5) are expressed relative to the infectious titers of HCVpp or of control VSV-Gpp particles determined on wt SR-BI-expressing SK-Hep1-CLDN1 cells (input ca. 10^4^ GFP iu), set to 100. (C) Results of HCVpp infection-enhancement induced by HDL (6 µg/ml cholesterol-HDL), expressed as ratios between average infectious titers determined in the presence or absence of HDL (mean±SD; n = 5). No changes of infectivity of VSV-Gpp control particles were detected under these experimental conditions (data not shown), as reported previously [25].
(0.13 MB TIF)

Protocol S1 Supplementary Materials and Methods
(0.03 MB DOC)
