# Supplementary figures and images for: Receptor Complementation and Mutagenesis Reveal SR-BI as an Essential HCV Entry Factor and Functionally Imply Its Intra- and Extra-Cellular Domains
Source: PLoS Pathog. 2009 Feb 20;5(2):e1000310. doi: 10.1371/journal.ppat.1000310 (PMC2636890; doi:10.1371/journal.ppat.1000310)

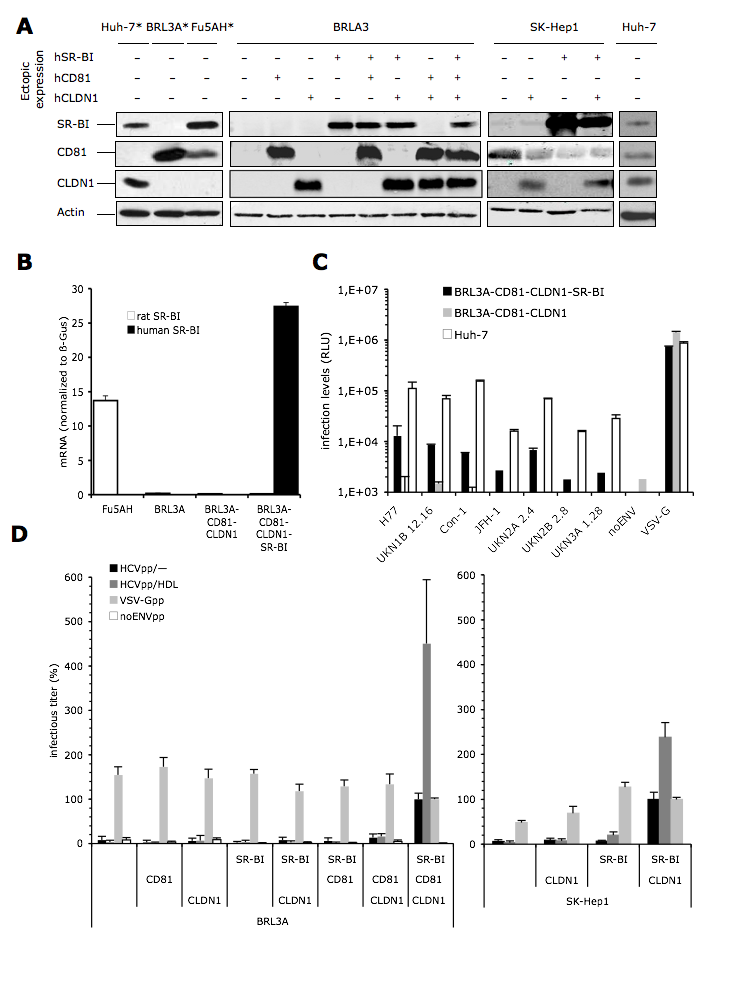

Supplement: Figure S1 — HCVpp entry in receptor-complemented BRL3A or SK-Hep1 cells. (A) Western blot analysis of the indicated HCV receptors in lysates of BRL3A, SK-Hep1, and Huh-7 cells ectopically expressing (+) or not expressing (−) the indicated HCV receptors using SR-BI (CLA-1, BD Bioscience), CD81 (JS81, Pharmingen), and CLDN1 (mouse anti-Claudin-1, Interchim) antibodies. The endogenous rat orthologs of these molecules were detected in BRL3A, Fu5AH rat hepatoma cells, and Huh-7 cells (Huh-7*, Fu5AH*, and BRL3A*) by Western blot analysis using cross-reactive antibodies against SR-BI (400-104, Novus), CD81 (EAT-2, Santa Cruz Biotechnology), and CLDN1 (mouse anti-Claudin-1, Interchim) antibodies. The actin staining (mAb AC74, Sigma-Aldrich) was used to ensure equal input of cell lysates. (B) Abundance of rat (white bars) and human (black bars) SR-BI mRNA levels in Fu5AH, BRL3A, BRL3A-CD81-CLDN1, and BRL3A-CD81-CLDN1-SR-BI cells. Total RNA was then extracted, quantified by Real time quantitative PCR, and normalized to rat β-Gus housekeeping gene (Protocol S1). Expression data were corrected for PCR efficiencies of the target and the reference gene, thus making possible analysis of the expression of one gene relative to the others. (C) Results of HCV entry assays on BRL3A-CD81-CLDN1-SR-BI, BRL3A-CD81-CLDN1, and Huh-7 target cells using HCV pseudo-particles carrying a luciferase marker gene and harboring E1E2 glycoproteins derived from the indicated genotypes/subtypes 1a (H77), 1b (Con-1, UKN1B 12.16), 2a (JFH-1, UKN2A 2.4), 2b (UKN2B 2.8), and 3a (UKN3A 1.28), as indicated [15], control viral particles harboring the VSV-G glycoprotein (diluted 1/100) or no glycoprotein (noENV). Results display average infectious titers, expressed as Luciferase unit (RLU) per 105 target cells (mean±SD; n = 3). (D) Results of HCV entry assays on BRL3A (left panel) and SK-Hep1 (right panel) cells ectopically expressing the indicated HCV receptors using HCV pseudo-particles harboring H77-E1E2 glycoproteins ( [file ppat.1000310.s001.tif]

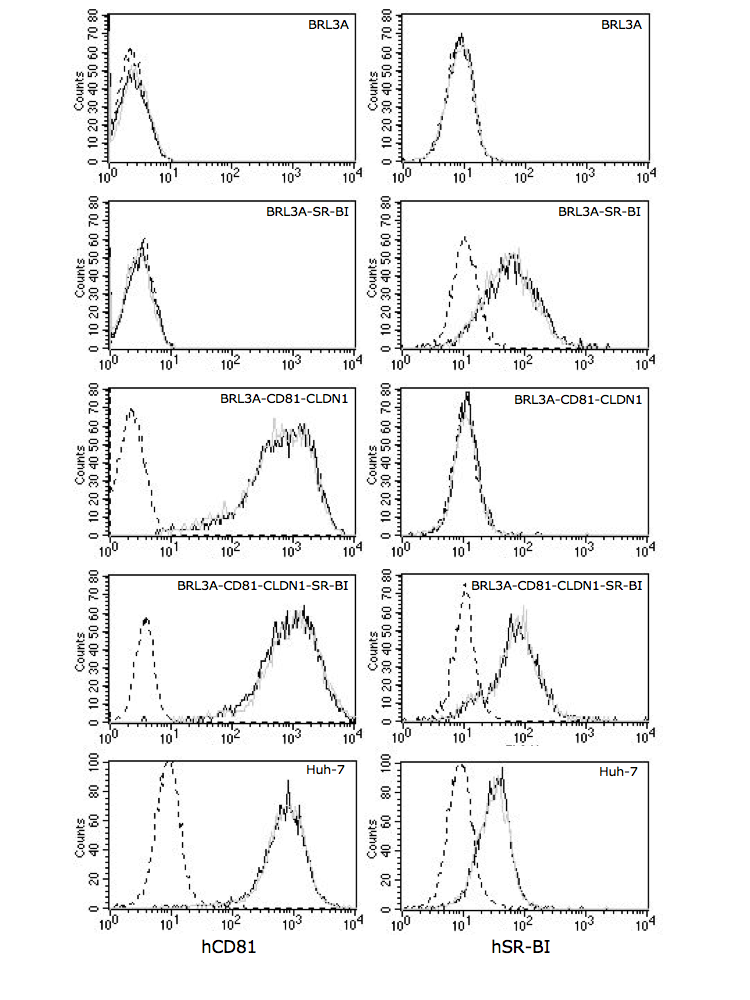

Supplement: Figure S2 — Cell surface expression of hCD81 and/or hSR-BI in BRL3A cells. BRL3A cells expressing the indicated entry factors were pre-incubated for 2 hrs in low serum-containing medium (0.1%), then incubated for 1 hr at 37°C in the absence (black lines) or in the presence (gray lines) of HDL (6 µg/ml cholesterol-HDL) before staining with JS81 (left panels) or CLA-1 (right panels) antibodies. The background of fluorescence was provided by staining the cells with the secondary antibodies only (dotted lines). (0.37 MB TIF) [file ppat.1000310.s002.tif]

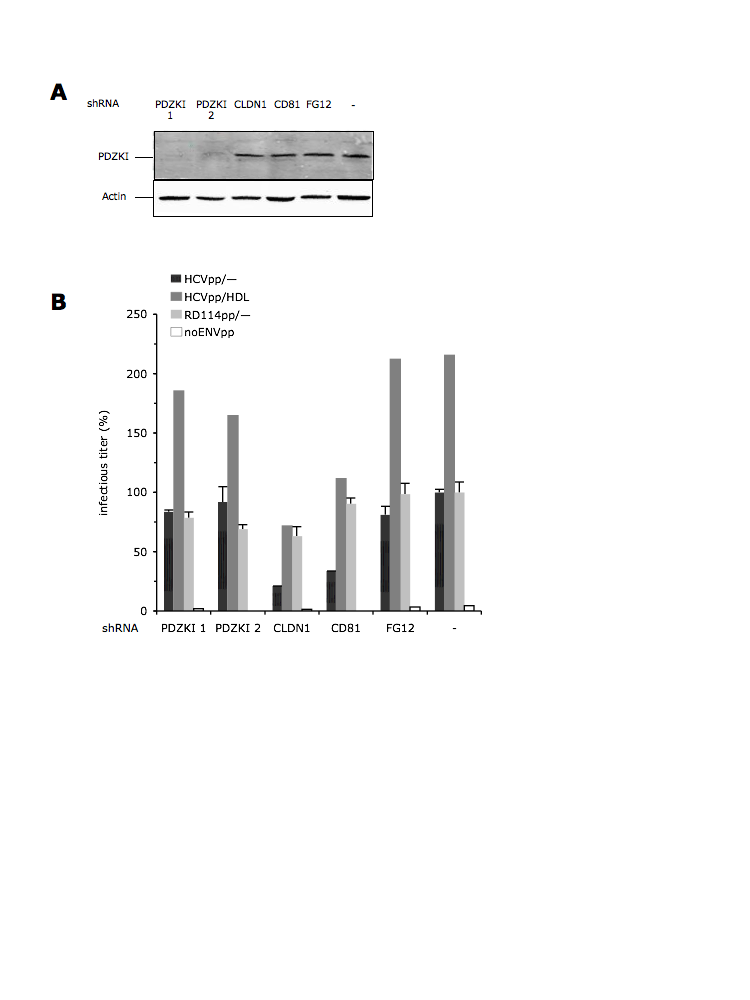

Supplement: Figure S3 — HCV entry in PDZK1 down-regulated target cells. PDZK1 (GenBank accession number: NM_002614) down-regulation was induced upon expression of specific shRNAs (PDZK1-1: 5′-GCTATGGCTTTCACTTAAAT and PDZK1-2: 5′-GAAAGAAGGCCTATGATTA) via the FG12 lentiviral vector [84] introduced in Huh-7 target cells. As controls, Huh-7 cells were transduced with FG12-derived vectors carrying shRNAs for CLDN1 (5′-AAGTGCTTGGAAGACGAT) and CD81 (5′-GATCGATGACCTCTTCTCC) or were left intact (−). (A) Western blot analysis of PDZK1 in lysates of Huh-7 cells in which these shRNAs were expressed (rabbit polyclonal Ab NB 400-1491, Novus Biologicals). The actin staining (mAb AC74, Sigma-Aldrich) was used to assess cell density. (B) Results of HCV entry assays on Huh-7 cells expressing PDZK1 or control shRNAs using HCV pseudo-particles harboring H77-E1E2 glycoproteins (HCVpp), control viral particles harboring the RD114 glycoprotein (RD114pp), or no glycoprotein (noENVpp). The viral particles, containing a CD90 marker gene, were produced in cell culture media devoid of serum lipoproteins. The results of infectivity (mean±SD; n = 3) are expressed relative to the infectious titers of HCVpp or of control RD114pp determined on intact Huh-7 cells, which were determined 72 hr after infection by measuring CD90 reporter gene expression by FACS analysis using an allophycocyanin (APC)-conjugated anti-CD90 mAb (clone 5E10, BD Pharmingen). As indicated, HCVpp entry assays were performed in the absence (−) or in the presence (HDL) of 6 µg/ml cholesterol-HDL. (0.09 MB TIF) [file ppat.1000310.s003.tif]

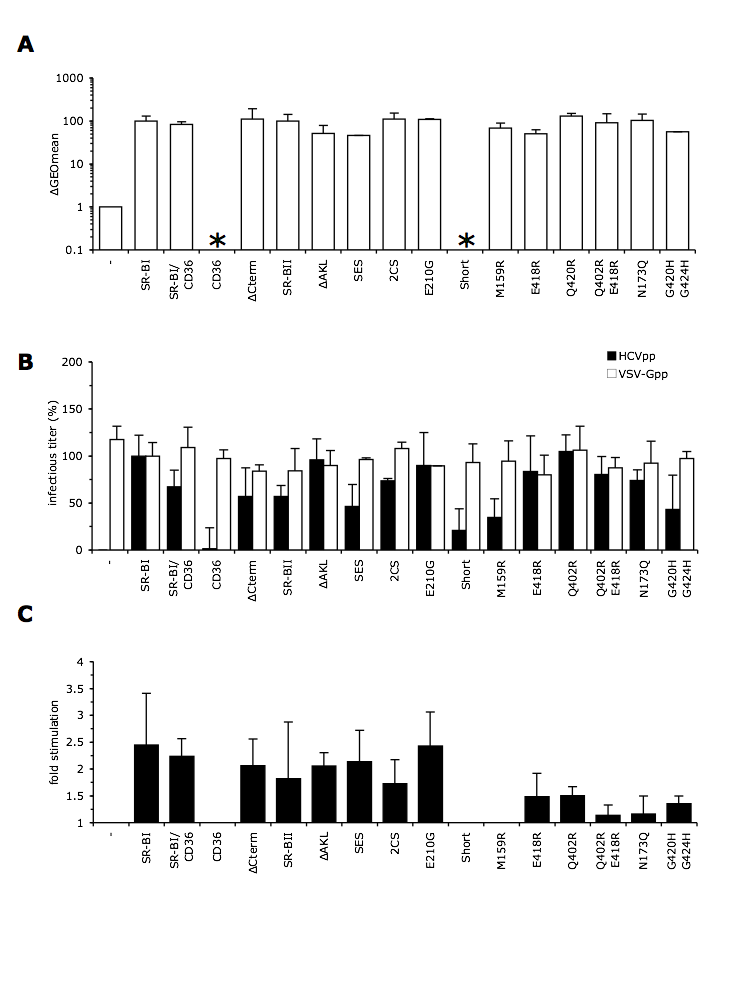

Supplement: Figure S4 — HCVpp entry in SK-Hep1-CLDN1 cells expressing SR-BI mutants. (A) Cell surface expression (white bars) of SR-BI mutants/isoforms as determined using anti-SR-BI antibody (CLA-1, BD Bioscience). The results of cell surface expression, analyzed by flow cytometry of SK-Hep1-CLDN1 cells transduced with retroviral vectors carrying the indicated SR-BI mutants, are expressed as the average percentages of GEOmean (geometric mean) fluorescence shifts (mean±SD; n = 3) detected between mutant receptor-expressing cells and parental (−) cells, relative to cells expressing wild-type SR-BI (ca. 40-fold GEOmean shift, Figure 1A) set to 100. Cell surface expression of CD36 (*, data not shown) was verified using a CD36 antibody (FA6-152, abcam). Cell surface expression of SR-BI-Short (*, data not shown) was verified by immuno-blotting using an antibody against SR-BI C-terminus (400-104, Novus) on surface-biotinylated proteins that were purified with streptavidin-coated beads. (B) Effect of SR-BI mutations on infectivity of HCVpp produced in serum-free media. The results of infectivity (mean±SD; n = 5) are expressed relative to the infectious titers of HCVpp or of control VSV-Gpp particles determined on wt SR-BI-expressing SK-Hep1-CLDN1 cells (input ca. 104 GFP iu), set to 100. (C) Results of HCVpp infection-enhancement induced by HDL (6 µg/ml cholesterol-HDL), expressed as ratios between average infectious titers determined in the presence or absence of HDL (mean±SD; n = 5). No changes of infectivity of VSV-Gpp control particles were detected under these experimental conditions (data not shown), as reported previously [25]. (0.13 MB TIF) [file ppat.1000310.s004.tif]
