# Supplementary material for: Receptor Complementation and Mutagenesis Reveal SR-BI as an Essential HCV Entry Factor and Functionally Imply Its Intra- and Extra-Cellular Domains
Source: PLoS Pathog. 2009 Feb 20;5(2):e1000310. doi: 10.1371/journal.ppat.1000310 (PMC2636890; doi:10.1371/journal.ppat.1000310)
Supplement: Protocol S1 — Supplementary Materials and Methods (0.03 MB DOC) [file ppat.1000310.s005.doc]

**Receptor complementation and mutagenesis reveal SR-BI as AN essential HCV entry factor and functionally imply its intra- and extra-cellular domains**

Marlène Dreux^1^, Viet Loan Dao Thi^1,*^, Judith Fresquet^1,*^, Maryse Guérin^2^, Zélie Julia^2^, Géraldine Verney^1^, David Durantel^3^, Fabien Zoulim^3^, Dimitri Lavillette^1^, François-Loïc Cosset^1,**^,**^#^** and Birke Bartosch^1,**^

^1^Université de Lyon, UCB-Lyon1, IFR128, Lyon, F-69007, France ; INSERM, U758, Lyon, F-69007, France ; Ecole Normale Supérieure de Lyon, Lyon, F-69007, France. ^2^INSERM, U551, Paris, F-75651, France. ^3^Université de Lyon, UCB-Lyon1, IFR62, Lyon, F-69008, France ; INSERM, U871, Lyon, F-69008, France; Hospices civils de Lyon (HCL), Lyon, F-69008, France.

* These authors contributed equally to this work

** These authors contributed equally to this work

**^#^** E-Mail: [flcosset@ens-lyon.fr](mailto:flcosset@ens-lyon.fr)

**Supplementary information - Supplementary Materials and Methods.**

**RNA extraction, reverse-transcription and quantitative-PCR**. Cells were cultured in 6-well plates and incubated at 37°C until confluence. Cells were then washed twice with cold PBS and total RNA was extracted using a NucleoSpin RNA II kit (Macherey-Nagel) according to the manufacturer's instructions. Then, 1500 ng of RNA was reverse transcribed with 75 ng of random hexamer using 200 units of M-MLV reverse transcriptase. An initial denaturation step for 5 mn at 68°C was followed by an elongation phase of 1 h at 42°C; the reaction was completed by a 5-mn incubation at 68°C.

Real time quantitative PCR was performed using a LightCycler LC480 (Roche). The reaction contained 2.5 ng of reverse transcribed total RNA, 150 pmol of forward and reverse primers (SR-BI: 5' GCT GCG CTC GGC GTT GTC AT 3' and 5' GGG ACG GGG ATC TCC TTC CA 3'; Cla-1: 5' GAG CTT TGG CCT TGG TCT ACC T 3' and 5' TCT TGT GCT CAC TCC ATT GTT TTC 3') and 5μl of Master Mix SYBR-Green, in a final volume of 10μl. Samples underwent the standard PCR protocol. Crossing Point (CP) values for genes of interest were normalized to the rat housekeeping gene Beta-GUS using forward primer (5'- AAG GGG ATC TTC ACT CGA CA-3') and reverse primer (5'- AAG GGG ATC TTC ACT CGA CA-3'). Expression data were based on the crossing points calculated with the software for LightCycler data analysis and corrected for PCR efficiencies of the target and the reference gene.
